# Climate change anxiety among psychiatric outpatients: associations with psychiatric symptoms and experience with climate change and natural disasters

**DOI:** 10.3389/fpsyt.2026.1874447

**Published:** 2026-07-24

**Authors:** So Hye Jo, Bon Hoon Koo, Eun-Jin Cheon, Seok-Ho Yun

**Affiliations:** Department of Psychiatry, Yeungnam University College of Medicine, Daegu, Republic of Korea

**Keywords:** anxiety symptoms, climate change anxiety, disaster exposure, psychiatric outpatients, resilience, somatic symptoms

## Abstract

**Background:**

Climate change anxiety (CCA) has emerged as a significant psychological response to the global climate crisis. While prior research focuses on general populations, evidence derived from psychiatric clinical samples remains scarce. This study aims to investigate CCA levels and their associations with psychiatric symptoms, resilience, and disaster-related experiences among psychiatric outpatients.

**Methods:**

A cross-sectional survey was conducted on 528 psychiatric outpatients aged 18–64 years at a university hospital in South Korea. The participants completed the Korean version of the Climate Change Anxiety Scale, along with standardized measures of depression (Patient Health Questionnaire-9), anxiety (General Anxiety Disorder-7), somatic symptoms (Korean Version of Somatic Symptom Scale-8), and resilience (Brief Resilience Scale). CCA was analyzed as a continuous variable, and a high-CCA group was defined as participants in the top quartile. Group comparisons, correlation analyses, and hierarchical regression analyses were performed.

**Results:**

The mean CCA score was 19.05 (standard deviation = 8.11), with 24.1% of the participants classified as exhibiting high levels of CCA; these elevated levels were significantly associated with greater depressive (*r* = 0.31), anxiety (*r* = 0.36), and somatic symptoms (*r* = 0.39) and lower resilience (*r* = −0.11). Patients with direct exposure to natural disasters reported significantly higher levels of CCA and greater psychiatric symptom severity, along with lower levels of resilience. In hierarchical regression analyses, somatic symptoms (β = 0.252, *p* < 0.001), anxiety (β = 0.194, *p* < 0.001), middle age (β = 0.194, *p* < 0.001), and cumulative disaster exposure (β = 0.133, *p* = 0.003) were significant factors associated with CCA, explaining 20.6% of variance.

**Conclusion:**

CCA among psychiatric outpatients is closely associated with broad emotional and somatic symptom burdens and decreased resilience. Instead of representing an isolated environmental concern, CCA may reflect underlying affective and physiological vulnerabilities exacerbated by cumulative exposure to climate-related stressors. These findings underscore the clinical relevance of climate-related distress and highlight the need for longitudinal studies and targeted interventions among vulnerable populations.

## 1 Introduction

April 22 was designated as Earth Day in 1970, marking 57 years since its establishment. Globally, the frequency and intensity of extreme weather events (e.g., heat waves, prolonged droughts, wildfires, and floods) have been steadily increasing. In recent decades, the frequency of climate-related disasters has risen sharply, increasing from approximately 340 incidents per year in 2000 to 665 in 2023. Consequently, the public’s exposure to environmental stressors has also significantly increased (1). Thus, climate change is no longer an uncertain threat in the distant future; it has transformed into a real-world disaster that directly threatens daily life and survival (2). As humanity experiences natural disasters and extreme weather events, directly or indirectly, evidence has clearly demonstrated that the climate crisis not only affects physical health and socioeconomic foundations but also exerts severe negative impacts on mental health (3). Consequently, awareness of and concern regarding climate change have also been increasing worldwide, and a significant number of people have experienced psychological anxiety due to the climate crisis.

One of the most prominent psychological consequences of climate change is “climate change anxiety (CCA)” or “eco-anxiety”. The American Psychological Association defines it as a chronic fear of environmental catastrophe and describes it as a persistent state of anxiety stemming from the recognition of the irreversible impacts of climate change and concerns for future generations (4). CCA has recently garnered global attention and emerged as a major mental health concern (5) CCA is a complex concept that extends beyond mere worry to encompass cognitive, emotional, and behavioral responses; it can be accompanied by persistent worry, feelings of helplessness, sleep disturbances, difficulty in concentration, and impaired functioning, among others (6–8). Alternatively, although anxiety can be interpreted as a normal and adaptive response to actual threats (2, 9), it can escalate to excessive or overwhelming levels due to the nature of a problem, which may be difficult to control and unlikely to be resolved through personal effort alone (10). In these cases, it can trigger physical symptoms such as persistent worry, sleep disturbances, and changes in appetite, and may further cause serious impairment in an individual’s daily life and cognitive and social functioning.

To date, research on CCA has primarily focused on the general adult population. According to previous studies, many people worldwide—including young adults and adolescents—report significant levels of anxiety and helplessness regarding climate change (10, 11). Scholars have reported that CCA exhibits significant positive correlations with depression, generalized anxiety disorder, suicidal behavior, and stress levels (12–17). However, studies that directly measure CCA and analyze its clinical characteristics among patients with mental disorders remain scarce.

Recent studies cautiously proposed that patients with preexisting mental illnesses may suffer greater psychological distress compared with the general population when exposed to climate change and ecological threats. Mental illness serves as a vulnerability factor that decreases one’s resilience and psychological buffering capacity against environmental stressors. Furthermore, many psychotropic medications, such as antipsychotics and antidepressants, impair thermoregulation, thereby sharply increasing physical risks during climate disasters such as heat waves (18). Furthermore, patients with lethargy associated with depression, the hyperarousal of anxiety disorders, and chronic somatic symptoms may experience greater distress due to the lack of cognitive and behavioral resources essential for coping with and adapting to sudden climate changes or disaster scenarios. The literature reported that individuals with preexisting anxiety or depressive disorders are approximately twice as likely to experience climate-related emotional distress (19), while climate-related disasters are closely associated with the onset and exacerbation of conditions such as posttraumatic stress syndrome, depression, anxiety, and substance use disorders (20). People with preexisting mental illnesses, particularly psychosis, dementia, and substance abuse, have a 2–3 times higher risk of death during heat waves (21–23). Globally, rising temperatures have been associated with an increased risk of hospitalization due to experiences diagnosed as bipolar disorder; schizophrenia; alcohol and substance abuse; and dementia, as well as a history of self-harm (18, 24–26). Furthermore, individuals with mental illnesses may be more vulnerable to complex stressors, such as climate change, due to impaired emotional regulation, increased sensitivity to threat, and lack of coping resources. During climate-related disasters, the risk of mental health deterioration may increase even further due to factors such as reduced access to healthcare and diminished social support (27).

Despite theoretical warnings, such as the abovementioned ones, highlighting that people with mental illness may be the most vulnerable to climate change, empirical studies that objectively measure and assess CCA among this population within actual clinical settings are very rare. The majority of studies are limited to healthy adults, college students, or groups of environmental activists. Therefore, a significant research gap exists regarding the perceptions of patients actually suffering from mental pathologies such as depression, anxiety, and somatic symptoms with regard to climate change, including relationships between actual clinical symptoms and CCA.

Therefore, the current study aims to assess the levels of CCA experienced by psychiatric outpatients and conduct an integrated analysis of these data alongside existing psychological scales (for depression, anxiety, somatic symptoms, and resilience), sociodemographic factors, and actual disaster experiences. By doing so, it intends to elucidate CCA as an environmental concern and a significant psychological phenomenon that must be considered within the psychiatric clinical domain. It also aims to provide foundational data for the development of future clinical assessments and psychosocial intervention strategies.

## 2 Materials and methods

This single-center, cross-sectional study was conducted at the Yeunggnam University Medical Center in Daegu, South Korea. This cross-sectional survey study was conducted from September 2025, to January 2026, on adults aged 18 to 64 years who were outpatients in the Department of Psychiatry. The participants were approached during their stay in the clinic’s waiting room and invited to the study. They were screened based on the inclusion and exclusion criteria after undergoing evaluation by a child and adolescent psychiatrist. Prior to completing the questionnaires, they were briefed on the purpose and content of the study and voluntarily provided written informed consent to participate. They completed a self-administered structured questionnaire during outpatient visits to the Department of Psychiatry to assess demographic information(age, sex, region, education, employment status, income, subjective economic status, marital status), direct and indirect experiences with climate change and natural disasters and psychological status (i.e., depression, anxiety, somatic symptoms, and resilience). If necessary, the researchers provided explanations and assistance. Each questionnaire lasted 10–15 minutes, and the participants received no compensation.

The inclusion criteria were defined as patients who received a diagnosis of one or more of the following conditions (e.g., mood, anxiety, stress-related, somatization, schizophrenia spectrum, and personality disorders) from a psychiatrist following the criteria of the Diagnostic and Statistical Manual of Mental Disorders, Fifth Edition (DSM-5). Moreover, they should have had undergone treatment for at least one month. The study included individuals who could understand the researcher’s explanation and voluntarily provide written informed consent. The exclusion criteria included patients with the inability to communicate or comprehend the questionnaire and complete it independently; a Mini-Mental State Examination score less than 20 or severe cognitive impairment, intellectual disability, or language disorders that prevent communication; acute psychosis or severe behavioral disturbances; those posing a persistent risk of self-harm or harm to others; and a history of significant organic brain disease or a physical crisis within the past three months.

For diagnostic group classification, each participant was assigned to one of six mutually exclusive categories based on the primary clinical diagnosis: depressive disorders, anxiety disorders, schizophrenia/bipolar disorders, trauma- and stress-related disorders, neurodevelopmental disorders, and other psychiatric disorders. The primary diagnosis was defined as the diagnosis that represented the main reason for the current outpatient treatment. Comorbid diagnoses were not counted as separate categories to avoid duplicate classification. The category of other psychiatric disorders included less frequent primary diagnoses that did not fall into the five major diagnostic groups, including sleep disorders, eating disorders, alcohol use disorder, personality disorders, and other less common conditions.

The Institutional Review Board (IRB) of Yeungnam University Hospital approved the study protocol (IRB No. 2025-08-029).

### 2.1 Measurement scales

#### 2.1.1 Demographic statistics

Data were collected on the participants’ age, gender, residential area, level of education, income, marital status, living situation (alone versus with others), level of social support (“is there someone you can turn to for comfort or help when you’re having a hard time?”), employment status, and experience with natural disasters (e.g., heat waves, floods, wildfires, and typhoons) or climate change (e.g., rising temperatures, prolonged droughts, excessive rainfall, and abnormal seasonal changes) within the past 2 years. The questionnaire on experiences with disasters and climate change assessed whether respondents had experienced climate-related events, but did not evaluate the severity of exposure, subjective sense of threat, personal loss, or trauma-related symptoms.

#### 2.1.2 Climate change anxiety scale

The Climate Change Anxiety Scale (CCAS) is a validated 13-item instrument for assessing psychological responses to climate change within the general adult population (8). It consists of a cognitive impairment subscale (8 items measuring intrusive thoughts and emotional responses) and a functional impairment subscale (5 items assessing interference with social and occupational activities due to climate-related anxiety). Items are rated using a five-point Likert-type scale. A higher score indicates a higher level of CCA, with scores ranging from 13 to 65. In related follow-up studies, a cutoff score of 21 or higher indicated mild-to-moderate CCA, and a score of 23 marked severe levels. Therefore, the current study adopted these scores as reference points (28). For descriptive purposes, participants were classified according to previously proposed reference cut-off scores (low CCA, <21; mild-to-moderate CCA, 21–22; and severe CCA, ≥23). Furthermore, it used the Korean version of the scale (K-CCAS), with established validity and reliability (29). Its correlation coefficient ranges from 0.3 to 0.5, while its reliability—measured using Cronbach’s α—is 0.91, indicating high internal consistency.

#### 2.1.3 Patient health questionnaire-9

The Patient Health Questionnaire 9 (PHQ-9), developed by Kroenke, Spitzer, and Williams, is a self-report diagnostic tool that consists of nine items used by clinical professionals to screen for, diagnose, monitor the course of, and measure the severity of depression (30). This tool assesses symptoms in the past 2 weeks based on the criteria of the DSM-4. High total scores indicate high levels of depression, with scores ranging from 0 to 27. Scores of 5, 10, 15, and 20 represent the cutoff points for mild, moderate, moderate-to-severe, and severe levels of depression, respectively. It has been adapted into Korean, with a Cronbach’s α coefficient of 0.81. Furthermore, correlations between each item and the total score were statistically significant (*r* = 0.28–0.70, *p* < 0.01).

#### 2.1.4 General anxiety disorder-7

The Generalized Anxiety Disorder-7 (GAD-7) is a seven-item assessment tool for measuring or evaluating the severity of generalized anxiety disorder (31), in which respondents rate the severity of their symptoms over the past 2 weeks. A GAD-7 score is calculated by assigning 0, 1, 2, and 3 points to the response categories “None,” “A few days,” “More than half the time,” and “Almost every day,” respectively, and summing the scores. The total GAD-7 score ranges from 0 to 21 points. Scores of 5, 10, and 15 represent the cutoff points for mild, moderate, and severe anxiety, respectively.

#### 2.1.5 Korean version of the somatic symptom scale-8

The Korean version of the Physical Symptom Scale-8 (K-SSS-8) is a validated Korean version of a brief, self-report questionnaire that consists of eight items designed to assess the burden of physical symptoms (32). This abbreviated questionnaire aids clinical assessment by measuring the degree of discomfort caused by general physical symptoms (e.g., pain, fatigue, and gastrointestinal problems) over the past 7 days. It demonstrates high internal consistency (Cronbach’s α) and test–retest reliability (*r* = 0.777).

#### 2.1.6 Brief resilience scale

The Brief Resilience Scale (BRS) was developed to assess the ability to recover from stressful situations (33). The scale consists of six items rated using a five-point Likert-type scale (1 = Strongly disagree, 5 = Strongly agree), yielding a total score ranging from 6 to 30. After summing the scores, the total is divided by 6 to calculate the mean score (range, 1.0–5.0). High scores indicate greater resilience. Scores of 1.0–2.9, 3.0–4.30, and 4.31–5.0 are interpreted as low, moderate, and high, respectively. The current study used the Korean version of the scale (34).

### 2.2 Statistical analysis

Prior to analysis, data quality was examined. Participants who failed to complete the questionnaire or with more than 10% of missing responses on key scales were excluded. In addition, responses exhibit straight-lining patterns (identical answers across the majority of items) were excluded. A total of 528 survey responses were analyzed. Depending on the questionnaire items, up to 27 responses were identified as having low reliability due to missing ratings or invariant responses. Overall, the proportion of missing data was less than 5%, which indicates minimal missingness. Given that the missing responses were deemed random, cases with missing values were excluded from the analyses on relevant variables. Statistical analyses were performed using IBM SPSS version 26.0 (IBM Corp., Armonk, NY, USA). Continuous variables were expressed as mean ± standard deviation (SD), while categorical variables were reported as frequencies and percentages. Descriptive statistics were used to describe demographic data. Moreover, group differences were analyzed using independent *t*-test, analysis of variance test, and chi-square test, as appropriate. For group comparison analyses, the high CCA group was operationally defined as participants in the upper 25% of the CCA total score distribution, whereas the remaining participants were classified as the lower CCA group. In the present sample, this upper-quartile criterion corresponded closely to a CCA total score of ≥23. Pearson’s correlation and hierarchical regression analyses were performed to identify and verify relationships, respectively, among factors associated with CCA. A difference with *p*-value <0.05 was considered statistically significant. Multicollinearity among independent variables was assessed using variance inflation factors (VIFs). A VIF value below 5 was considered to indicate the absence of problematic multicollinearity.

## 3 Results

### 3.1 Demographic characteristics

During the study period, a total of 1,347 eligible adult psychiatric outpatients were approached and invited to participate in the survey. Of these, 528 agreed to participate and completed the questionnaire, corresponding to a response rate of 39.2%. The remaining 819 patients declined participation. No participants were excluded from the overall analytic sample after survey completion. However, depending on the questionnaire items, up to 27 responses were identified as having missing ratings or invariant response patterns. Therefore, the final analytic sample consisted of 528 adult psychiatric outpatients. **Table 1** presents sociodemographic information, history of experiences with natural disasters or climate change, and diagnostic characteristics.

**Table 1:** Sociodemographic and clinical characteristics of the participants.

Variable	Group	n	%
Age group (yr)	Younger (19–39)	261	49.4
	Middle-aged (40–64)	240	45.5
Gender	Male	249	48.5
	Female	264	51.5
Region	Metropolitan city	372	72.4
	Small- or medium-sized city	94	18.3
	Rural area	48	9.3
Education	No or elementary school	3	0.6
	Middle school	16	3.1
	High school	225	43.8
	College	85	16.5
	University or higher	185	36.0
Employment status	Unemployed	140	27.3
	Student	95	18.5
	Employee	123	23.0
	Homemaker	71	13.8
	Self-employed	41	8.0
	Retired and others	43	8.4
Income (million KRW)	< 1	88	17.3
	1–3	121	23.8
	3–5	104	20.4
	> 5	103	20.2
	Refused to answer	93	18.3
Subjective economic status	Difficult	224	43.4
	Average	206	40.0
	Comfortable	85	16.5
Marital status	Single	315	60.6
	Married or cohabiting	191	36.8
	Divorced or widowed	14	2.7
Climate changes and Disasters	Direct experience	417	79.0
	Indirect experience	42	8.0
	No experience	63	11.9
Diagnostic group	Depressive disorders	165	31.3
	Anxiety disorders	58	11.0
	Schizophrenia/bipolar disorders	136	25.8
	Trauma- and stress-related disorders	79	15.0
	Neurodevelopmental disorders	38	7.2
	Other psychiatric disorders	52	9.8

Percentages are based on valid responses due to missing data for certain variables.

### 3.2 Psychological characteristics of the participants

**Table 2** presents the descriptive statistics on the psychological characteristics of the participants. The mean total score for CCA was 19.05 (SD = 8.11). In terms of the subscales, the mean scores for the cognitive and functional domains of CCA were 11.99 (SD = 5.23) and 7.06 (SD = 3.47), respectively. The group with low levels of CCA (below; <21) was the largest at 69.7% (n = 368); the group with mild–moderate levels of CCA (mild–moderate; ≥21) accounted for 6.2% (n = 33); and the group with severe CCA (severe; ≥23) accounted for 24.1% (n = 127). In the present sample, the previously proposed severe CCA cut-off score of ≥23 closely corresponded to the upper quartile of the observed CCA total score distribution.

**Table 2:** Psychological characteristics of the participants.

Variable	M	SD	Cutoff (clinical interpretation)
CCAS-TOTAL	19.05	8.11	Subthreshold or low-level (<21)
CCAS-COG	11.99	5.23	
CCAS-FUN	7.06	3.47	
PHQ-9_TOTAL	18.00	7.62	Moderately severe depression (≥15)
GAD-7_TOTAL	14.57	5.78	Moderate anxiety (≥10)
K-SSS-8_TOTAL	18.56	7.66	Very high (≥16)
BRS_MEAN	2.62	0.92	Low resilience (1.00–2.99)

Descriptive statistics and cutoff criteria of psychological variables.

M, mean; SD, standard deviation; CCA, climate change anxiety; CCA_TOTAL, total score in the CCAS; CCA-COG, cognitive impairment subscale of the CCAS; CCA-FUNC, functional impairment subscale of the CCAS; PHQ-9, Patient Health Questionnaire-9; GAD-7, Generalized Anxiety Disorder-7; K-SSS-8, Korean version of the Somatic Symptom Scale-8; BRS, Brief Resilience Scale.

The mean score for depression was 18.00 (SD = 7.62), corresponding to the clinically moderate-to-severe range (≥15), while the mean score for anxiety reached 14.57 (SD = 5.78), corresponding to a moderate level of anxiety (≥10). Furthermore, the mean score for physical symptoms was 18.56 (SD = 7.66), corresponding to a very high level (≥16). The mean score for resilience reached 2.62 (SD = 0.92), which falls under the low category (1.00–2.99).

### 3.3 Experiences with climate change and natural disasters

The study examined participants’ exposure to natural disasters within the past year (e.g., heat waves, floods, wildfires, and typhoons) and climate change phenomena within the past 2 years (e.g., rising temperatures, prolonged droughts, excessive rainfall, and abnormal seasonal changes). Indirect experience refers to experiences not personally encountered but learned about through news reports, TV, or the Internet or through stories shared by family, friends, or people within the participants’ immediate circle.

A total of 417 participants (79%) reported having at least one direct experience of climate change or a natural disaster, while 42 (8.0%) reported only indirect experience, and 63 (11.9%) reported no experience. In terms of direct experiences with natural disasters, heatwaves (65.5%) were the most common, followed by typhoons (12.9%), wildfires (6.7%), and floods (3.4%). Conversely, for indirect experiences, wildfires (57.3%) yielded the highest proportion, followed by floods (49.9%), typhoons (38.5%), and heat waves (14.5%). For direct experiences with climate change, rising temperatures (70.9%) were reported most frequently, followed by seasonal anomalies (54.0%), excessive rainfall (30.2%), and prolonged drought (13.0%). In contrast, the proportion of indirect experiences with climate change was highest for prolonged drought (53.8%), followed by excessive rainfall (41.2%), seasonal anomalies (26.8%), and rising temperatures (16.9%).

### 3.4 Differences in climate change anxiety by demographic factors

To identify the characteristics of individuals with high levels of CCA, differences in sociodemographic characteristics between the top 25% and bottom 75% groups were analyzed based on the total scores for CCA (**Table 3**). The results revealed a significant difference in age group. Within the top 25% high-risk group, middle-aged adults accounted for 61.3% (n = 84), thereby outnumbering younger adults, with a small effect size (Cramér’s V = 0.142). In contrast, no significant differences were found based on high-risk status in terms of diagnostic criteria, gender, residential area, level of educational, employment status, income, subjective economic status, or marital status (*p* > 0.05).

**Table 3:** Results of chi-square analyses of sociodemographic differences by climate change anxiety scores.

Variable	χ^2^	df	*P*-value	Cramer’s V
Diagnostic group	6.35	5	0.274	0.115
Age group	9.64	1	0.002	0.142
Gender	0.93	1	0.629	0.044
Region	4.26	2	0.119	0.094
Level of education	2.92	6	0.819	0.078
Employment status	4.41	7	0.731	0.096
Income	3.28	4	0.513	0.083
Subjective economic status	3.47	4	0.483	0.085
Marital status	6.03	5	0.303	0.112

Independent samples *t*-tests were conducted. First, differences in levels of CCA were examined between the groups living alone and living with others; no significant difference was found in the scores of CCA between them (*t*(512) = −0.65, *p* = 0.514). Furthermore, differences in levels of CCA between the group with and without social support (help) were analyzed. No significant difference in the level of CCA was noted (*t*(517) = −0.63, *p* = 0.531). Moreover, no statistically significant differences were observed in the presence or absence of social support factors regarding the CCA-cognitive impairment subgroup (*t*(517) = −0.72, *p* = 0.470) and CCA-functional impairment subgroup (*t*(517) = −0.38, *p* = 0.705) (*p* > 0.05).

An independent samples *t*-test was also performed to verify differences in levels of CCA across age groups. **Table 4** presents the results. The scores for CCA were significantly higher for middle-aged adults compared with younger adults. Scores for CCA-cognitive impairment and CCA-functional impairment were also significantly higher for middle-aged adults than those for younger adults.

**Table 4:** Climate change anxiety by age group.

Variable	Age group	N	M (SD)	*t*	*P*-value	d
CCA_TOTAL	Younger	261	19.31 (10.99)	−3.831	0.001	0.49
	Middle-aged	240	24.56 (18.42)			
CCA-COG	Younger	261	12.34 (8.05)	−2.951	0.003	0.43
	Middle-aged	240	14.89 (10.94)			
CCA-FUNC	Younger	261	6.97 (4.33)	−4.391	0.001	0.50
	Middle-aged	240	9.67 (8.56)			

M, mean; SD, standard deviation; CCA_TOTAL, total score in the Climate Change Anxiety Scale; CCA-COG, Cognitive Impairment subscale; CCA-FUNC, Functional Impairment subscale.

### 3.5 Differences in climate change anxiety and psychological characteristics based on experience with climate change or natural disasters

To examine disaster experience, the participants were reclassified under the direct experience (n = 417, 79.9%) and indirect/no experience (n = 105, 20.1%) groups. In this study, direct experience pertains to at least one instance of personal exposure to a disaster.

To verify differences in CCA and mental health indicators according to the types of climate change and natural disaster, an independent samples *t*-test was conducted (**Table 5**). The results demonstrated that the scores for CCA were significantly higher for the direct experience group compared with those of the indirect/no experience group. Moreover, scores for depression (*p* = 0.034) and anxiety (*p* = 0.007) were significantly higher for the direct experience group. Scores for physical symptom were also higher for the direct experience group, with a statistically significant difference (*p* = 0.039). In contrast, scores for resilience were significantly lower for the direct experience group (*p* = 0.002).

**Table 5:** Climate change anxiety, depression, anxiety, and somatization by disaster experiences.

Variable	Type of disaster experience	M (SD)	*t* (df)	*P-*value	d
CCA	Direct	19.55 (8.02)	1.78 (167.20)	0.039	0.20
	Indirect/none	18.08 (7.39)			
PHQ-9	Direct	18.24 (7.56)	1.83 (154.79)	0.034	0.20
	Indirect/none	16.71 (7.58)			
GAD-7	Direct	14.84 (5.74)	2.49 (147.57)	0.007	0.28
	Indirect/none	13.25 (5.63)			
K-SSS-8	Direct	18.83 (7.42)	1.76 (137.12)	0.039	0.20
	Indirect/none	17.32 (8.44)			
BRS	Direct	2.75 (1.38)	−3.19 (520.00)	0.002	-0.26
	Indirect/none	3.26 (1.75)			

M, mean; SD, standard deviation; CCA, climate change anxiety; PHQ-9, Patient Health Questionnaire-9; GAD-7, Generalized Anxiety Disorder-7; K-SSS-8, Korean Somatic Symptom Scale-8; BRS, Brief Resilience Scale.

### 3.6 Differences in climate change anxiety across psychiatric diagnostic groups

To examine whether CCA differed by diagnostic group, one-way ANOVA was conducted across six diagnostic categories. No statistically significant differences were found among diagnostic groups in the CCA total scores. The difference in CCA total scores was not significant and showed a small effect size (F(5, 522) = 2.14, *p* = 0.060, η^2^ = 0.020). The same pattern was observed for the cognitive impairment subscale (F(5, 522) = 2.11, *p* = 0.063, η^2^ = 0.020) and the functional impairment subscale (F(5, 522) = 1.70, *p* = 0.132, η^2^ = 0.016). These effect sizes were small.

Descriptively, the depressive disorders group showed the highest mean CCA total score (M = 20.47, SD = 8.54), followed by the schizophrenia/bipolar disorders group (M = 19.30, SD = 8.76), trauma- and stress-related disorders group (M = 18.28, SD = 7.31), other psychiatric disorders group (M = 18.13, SD = 8.14), anxiety disorders group (M = 17.50, SD = 5.87), and neurodevelopmental disorders group (M = 17.24, SD = 7.58).

### 3.7 Association between key factors influencing climate change anxiety

Pearson correlation analysis was conducted to examine relationships among the key factors associated with CCA (**Table 6**). The results indicated that the total score for CCA was significantly positively correlated with depressive (*r* = 0.31, *p* < 0.001), anxiety (*r* = 0.36, *p* < 0.001), and somatic (*r* = 0.39, *p* < 0.001) symptoms. In contrast, a significant negative correlation was observed for resilience (*r* = −0.11, *p* < 0.05), demonstrating that high levels of resilience were associated with low levels of CCA. Additionally, the cumulative number of direct experiences with climate change and natural disasters yielded a significant positive correlation with the total score for CCA (*r* = 0.22, *p* < 0.001). Alternatively, sociodemographic variables such as income level (*r* = −0.03, *p* > 0.05), number of family members living together (*r* = −0.01, *p* > 0.05), and number of social support resources (*r* = −0.04, *p* > 0.05) did not exhibit significant correlations with the total score for CCA.

**Table 6:** Pearson correlations among variables.

Variable	1	2	3	4	5	6	7	8	9	10	11
1. CCA_TOTAL	–										
2. CCA-COG	0.96^***^	–									
3. CCA-FUNC	0.90^***^	0.73^***^	–								
4. PHQ-9	0.31^***^	0.29^***^	0.29^***^	–							
5. GAD-7	0.36^***^	0.33^***^	0.34^***^	0.82^***^	–						
6. K-SSS-8	0.39^***^	0.37^***^	0.35^***^	0.75^***^	0.76^***^	–					
7. BRS	−0.11^*^	−0.08	−0.13^**^	0.07	0.08	0.11^*^	–				
8. Disaster Exp.	0.22^***^	0.22^***^	0.18^***^	0.17^***^	0.21^***^	0.18^***^	−0.05	–			
9. Income	−0.03	−0.03	−0.02	−0.01	−0.03	−0.06	−0.01	−0.03	–		
10. Co-residents	−0.01	−0.01	0.00	0.04	0.04	0.05	−0.01	0.02	0.10^*^	–	
11. Sources of support	−0.04	−0.02	−0.07	−0.03	0.04	0.00	0.06	0.09	−0.01	−0.04	–

^*^ *p* < 0.05. ^**^p < 0.01. ^***^p < 0.001.

CCA_TOTAL, total score in the CCAS; CCA-COG, Cognitive Impairment subscale; CCA-FUNC, Functional Impairment subscale; PHQ-9, Patient Health Questionnaire-9; GAD-7, Generalized Anxiety Disorder-7; K-SSS-8, Korean Somatic Symptom Scale; BRS, Brief Resilience Scale; Disaster Exp., cumulative disaster exposure; Income, household income; Co-residents, number of co-residents; Sources of support, number of available sources of social support.

Hierarchical regression analysis was conducted to identify the factors associated with CCA (**Table 7**). Sociodemographic variables (i.e., age group, gender, and income) were included in Stage 1. The results indicated that the model was statistically significant (*F* = 4.38, *p* = 0.005), with an explanatory power of 2.9% (*R*^2^ = 0.029). At second stage, only age group (β = 0.169, *p* < 0.001) significantly associated CCA, while gender and income were not significant. Clinical symptom variables (i.e., depression, anxiety, somatic symptoms and resilience) were added in Stage 2. The explanatory power increased significantly by 16.0% (Δ*R*^2^ = 0.160, *p* < 0.001), which resulted in a total explanatory power of 18.9% (*R*^2^ = 0.189). At third stage, anxiety (β = 0.216, *p* = 0.010) and somatic symptoms (β = 0.270, *p* < 0.001) significantly predicted CCA, while depression and resilience were not significant. In Stage 3, the inclusion of cumulative disaster experience significantly increased the explanatory power by an additional 1.7% (Δ*R*^2^ = 0.017, *p* = 0.003), thereby resulting in a final model with an explanatory power of 20.6% (*R*^2^ = 0.206). In the final model, physical symptoms (β = 0.252, *p* < 0.001), anxiety (β = 0.194, *p* = 0.019), age group (β = 0.194, *p* < 0.001), and cumulative disaster experience (β = 0.133, *p* = 0.003) significantly predicted CCA, while gender, income, depression and resilience were nonsignificant. In an additional regression analysis including diagnostic group categories as covariates, the overall pattern of findings remained unchanged. Age group, anxiety symptoms, somatic symptoms, and cumulative disaster exposure remained significantly associated with CCA, whereas diagnostic group variables were not significant in the final model. Multicollinearity diagnostics showed that all VIF values were below 4.0 in the final regression model, indicating no evidence of problematic multicollinearity. The VIF values for PHQ-9, GAD-7, K-SSS-8, and BRS were 3.73, 3.71, 2.84, and 1.03, respectively.

**Table 7:** Results of the hierarchical regression analysis on factors predicting climate change anxiety.

Variable	*B*	95% CI	*SE*	β	*t*	*p*	VIF
Step 1							
(Constant)	16.47	[13.16, 19.77]	1.68	—	9.78	< .001	—
Age Group	2.57	[1.15, 3.99]	0.72	.17	3.56	< .001	1.02
Gender	−0.35	[−1.75, 1.04]	0.71	−.02	−0.50	.531	1.02
Income	−0.14	[−0.67, 0.38]	0.27	−.03	−0.54	.588	1.00
Model summary: *R*^2^ = .029, Δ*R*^2^ = .029, *F* = 4.38, *p* = .005							
Step 2							
(Constant)	7.87	[3.93, 11.80]	2.00	—	3.93	< .001	—
PHQ-9	−0.07	[−0.24, 0.10]	0.09	−.07	−0.79	.428	3.77
GAD-7	0.29	[0.07, 0.50]	0.11	.22	2.60	.010	3.68
K-SSS	0.27	[0.13, 0.42]	0.07	.27	3.71	< .001	2.83
BRS	−0.01	[−0.12, 0.11]	0.06	−.01	−0.16	.876	1.03
Model summary: *R*^2^ = .189, Δ*R*^2^ = .160, *F* = 21.40, *p* < .001							
Step 3							
(Constant)	6.74	[2.76, 10.71]	2.02	—	3.33	< .001	—
Age Group	2.95	[1.63, 4.27]	0.67	.19	4.38	< .001	1.07
Gender	−0.30	[−1.57, 0.97]	0.65	−.02	−0.46	.645	1.02
Income	0.03	[−0.45, 0.51]	0.24	.01	0.12	.908	1.01
PHQ-9	−0.06	[−0.22, 0.11]	0.09	−.06	−0.69	.494	3.73
GAD-7	0.26	[0.04, 0.47]	0.11	.19	2.35	.019	3.71
K-SSS	0.26	[0.11, 0.40]	0.07	.25	3.49	< .001	2.84
BRS	−0.01	[−0.12, 0.11]	0.06	−.01	−0.14	.888	1.03
Disaster Exp.	0.52	[0.18, 0.86]	0.17	.13	3.01	.003	1.07
Model summary: *R*^2^ = .206, Δ*R*^2^ = .017, *F* = 9.03, *p* = .003							

CCA, climate change anxiety; PHQ–9, Patient Health Questionnaire–9; GAD–7, Generalized Anxiety Disorder–7; K–SSS, Korean Somatic Symptom Scale; BRS, Brief Resilience Scale; Disaster Exp.= Cumulative number of direct disaster exposure experiences.

## 4 Discussion

Previous studies primarily focused on eco-anxiety among the general public. To the best of our knowledge, the current study is one of the pioneering studies to examine the clinical associations of CCA among psychiatric outpatients. The results indicate that CCA among psychiatric patients includes a high-risk subgroup, with middle-aged individuals exhibiting higher levels of CCA associated with direct experiences with climate change and disasters. In summary, CCA was positively associated with depression, anxiety, and somatic symptoms and negatively associated with resilience. The results of the hierarchical regression analysis revealed that the factors associated with CCA included somatic symptoms, anxiety, age group (middle-aged), and cumulative disaster experience. These findings underscore that CCA among psychiatric patients may be better interpreted as a manifestation of underlying affective and somatic vulnerabilities, instead of an isolated environmental concern.

### 4.1 Patterns of climate change anxiety among psychiatric patients: vulnerability in subgroups

A key contribution of this study is its identification of a high-risk subgroup (24.1%) experiencing severe CCA. These results demonstrate that CCA may not be uniformly distributed across the entire patient population but concentrated to specific vulnerable subgroups. Moreover, climate-related issues may serve as a new source of stress that exacerbates preexisting psychological vulnerabilities.

The mean total score for CCA for the patient group was 19.05 (SD = 8.11), indicating a normal or low level that does not reach a clinically significant level of CCA. When converted to an average score on a five-point scale, this score aligns with the results of studies on CCA among adults in Korea (35). Many previous studies reported average scores for anxiety among study populations, ranging from 1 to 2 on a five-point scale (36, 37). More than half of the participants exhibited low levels of CCA; a potential reason underlying this finding is that psychiatric outpatients are currently experiencing high levels of emotional distress, thus rendering climate-related anxiety as relatively less pronounced.

### 4.2 Differences in climate change anxiety by age

Middle-aged adults produced significantly higher total scores than did younger adults for CCA. Examining the subfactors, the study found that middle-aged adults yielded significantly higher scores than those of younger adults in the cognitive and functional impairment domains of CCA. This finding emphasizes that CCA tends to be higher overall for middle-aged adults. Previous studies reported that CCA is more commonly experienced by women (9, 35), younger individuals (38, 39), those with high levels of education (8), and low-income groups who are more exposed to environmental risks (40). In a nationally representative sample collected from a social survey conducted in Korea in 2024, scores for CCA were higher for middle-aged adults (40–59 years) and young adults (20–39 years) than for older adults (41).

In the current study, middle-aged adults (aged 40–64 years) exhibited significantly higher levels of anxiety than did young adults in the cognitive and functional impairment domains, thus reflecting the specific characteristics of this demographic. The high levels of anxiety observed for middle-aged adults may be caused by economic and social responsibilities. As the generation that bears responsibility for household finances, housing, and agriculture/fishery, they may likely perceive climate change events such as heatwaves, droughts, and wildfires as immediate, real-world threats that directly impact their livelihood. Additionally, they may exhibit a heightened perception of climate change and more experience with actual disasters or environmental changes. As a generation that has experienced previous climates and current extreme climate changes, they may perceive the severity of the climate crisis more acutely, thus leading to increased anxiety in the cognitive domain.

Psychiatric diagnosis and socioeconomic variables were not significantly associated with CCA, whereas age showed a significant association. This finding underscores that CCA does not merely pertain to socioeconomic support and resources; instead, it is mainly related to psychological processing involving emotions and cognition and may be linked to generational and life-cycle characteristics.

### 4.3 Relationship of experiences with climate change and natural disasters with climate change anxiety

In terms of direct experiences with natural disasters and climate change, heatwaves (65.5%) and rising temperatures (70.9%), respectively, were the most common. The regression analysis showed that cumulative disaster experiences were significantly associated with greater CCA. This finding suggests that repeated and cumulative disaster exposure may be one of the factors potentially associated with higher levels of CCA. This notion is further supported by the finding that the group with direct experience with disasters and climate change obtained significantly higher scores for CCA, higher levels of clinical symptoms, and lower levels of resilience compared with the group with indirect/no experience. Thus, the study infers that CCA among individuals with mental illness stems from actual trauma and suffering emerging from experiences that pose a direct threat to survival, instead of vague environmental concerns. This aspect demonstrates that exposure to climate change could become a significant factor for mental health amid the ongoing climate crisis. However, this association should be interpreted with caution. Rather than directly attributing CCA to posttraumatic reactions or trauma, repeated exposure to environmental threats may intensify CCA by increasing risk perception, future uncertainty, and a diminished sense of control.

### 4.4 Clinical factors associated with climate change anxiety

The explanatory power of the hierarchical regression analysis reached 20.6% (*R*^2^ = 0.206). In the final model, the variables that significantly predicted CCA were physical symptoms, anxiety, age, and cumulative disaster experience, while gender, income, and depression were nonsignificant. Although the sample included heterogeneous psychiatric diagnoses, additional analyses adjusting for diagnostic group did not materially alter the main findings. This supports the interpretation that CCA in psychiatric outpatients may be more closely related to transdiagnostic symptom dimensions, such as anxiety and somatic symptoms, than to categorical psychiatric diagnoses. Somatic symptoms showed the strongest association with CCA. In individuals with mental disorders, CCA is accompanied by hyperarousal of the autonomic nervous system and is experienced directly through the body. Therefore, it may be more closely associated with somatic anxiety than with cognitive worry. The participants’ average scores for somatic symptoms were notably high, indicating that individuals who are more sensitive to bodily sensations and experience symptoms more intensely may also perceive threats due to climate related changes more acutely and exhibit heightened anxiety.

However, this finding should be interpreted with caution. Although multicollinearity diagnostics showed no evidence of problematic multicollinearity, the PHQ-9, GAD-7, and K-SSS-8 are conceptually and empirically related measures of psychological distress. CCA may partly overlap with broader psychological distress, sharing features such as repetitive worry, threat sensitivity, negative affect, and somatic arousal. At the same time, CCA may be distinguished by its climate-specific cognitive and emotional content, including intrusive thoughts about climate change, concerns about environmental threats, and functional impairment related to climate concerns. Therefore, CCA in psychiatric outpatients may be best conceptualized as a climate-specific manifestation embedded within broader psychological distress, rather than as either an entirely independent phenomenon or a nonspecific reflection of psychiatric severity.

Although the present sample included heterogeneous psychiatric diagnoses, diagnostic group differences in CCA were not statistically significant and the effect sizes were small. These findings suggest that CCA among psychiatric outpatients may be better understood as a transdiagnostic phenomenon related to current symptom dimensions, particularly anxiety and somatic symptoms, and disaster-related experiences, rather than as a phenomenon confined to a specific psychiatric diagnostic category. However, because diagnostic classification was based on the primary clinical diagnosis, the potential influence of psychiatric comorbidity and detailed illness characteristics could not be fully examined.

### 4.5 Resilience and social support

In the correlation analysis, income level, number of cohabiting family members, and number of sources of social support were not correlated with the relationships among the key factors associated with CCA. In summary, CCA displayed positive correlations with depression, anxiety, and somatic physical symptoms and a negative correlation with resilience. However, resilience was not independently associated with CCA in the final regression model after adjusting for depressive symptoms, anxiety symptoms, somatic symptoms, and cumulative disaster exposure. Therefore, the association between resilience and CCA should be interpreted cautiously. Rather than indicating an independent protective effect, lower resilience may reflect broader psychological distress or reduced coping resources among psychiatric outpatients.

The difference between the results of the current and previous studies indicates that CCA may be more closely associated with an individual’s emotional vulnerability, subjective perceptions, and experiences with climate change than with traditional socioeconomic indicators. However, the social support items used in this study primarily assessed the quantitative availability of support and may not have fully captured qualitative aspects of support, such as perceived emotional safety, trust, or attachment security. Therefore, the nonsignificant findings regarding social support should not be interpreted as evidence that social support is unimportant. Further studies employing a multidimensional approach are required.

Resilience is defined as an individual’s ability to overcome adversity, adapt positively, and achieve growth (42) Research on resilience reported that social support is a key protective factor, and forming and maintaining strong social bonds is beneficial (43, 44) The underlying reason is that, during difficult times, people seek emotional support and material assistance from those close to them such as family, friends, and neighbors. Previous scholars disaster studies have suggested high levels of social support during and after a disaster may buffer psychological distress after traumatic or stressful events (45). However, in the present cross-sectional study, resilience did not remain independently associated with CCA when clinical symptom variables were included. Future studies should examine whether resilience moderates or mediates the relationship between climate-related disaster exposure, psychiatric symptoms, and CCA. Clinically, interventions targeting anxiety and somatic hyperarousal may be particularly relevant, while resilience-enhancing approaches may be considered as a complementary strategy rather than as a primary implication of the present findings. Clinically, interventions targeting anxiety and somatic hyperarousal may be particularly relevant, while resilience-enhancing approaches may be considered as a complementary strategy rather than as a primary implication of the present findings.

### 4.6 Limitations

This study has several limitations. Because of the cross-sectional design, causal relationships or temporal sequences among psychiatric symptoms, disaster exposure, and CCA could not be determined. Second, the use of self-report scales could have increased recall and response biases. In particular, because participants were treatment-seeking psychiatric outpatients, their responses may have been influenced by current symptom burden, including emotional distress, anxiety, depressive symptoms, and somatic concerns. Thus, the findings should be interpreted within the context of psychiatric outpatient care and should not be generalized directly to the broader population. Third, the study was conducted within a single psychiatric outpatient setting, which may limit generalizability. Future multicenter studies including psychiatric outpatients from diverse regions and healthcare settings are needed to confirm the generalizability of the present results. Fourth, disaster experiences were measured primarily based on whether such experiences were direct and the cumulative number of occurrences. The questionnaire did not assess detailed characteristics of exposure, such as objective severity, subjective threat perception, personal losses, displacement, or trauma-related responses. In addition, the final regression model explained approximately 20% of the variance in CCA, suggesting that other unmeasured factors, such as media exposure, environmental attitudes, climate risk perception, and broader psychosocial variables, may also contribute to climate-related distress. Sixth, we did not employ structural equation modeling (SEM), which can account for measurement error and evaluate latent relationships among psychological constructs. Because the present study was designed as an exploratory investigation of factors associated with CCA, conventional regression-based analyses using validated scale scores were applied. Future studies may benefit from SEM approaches to clarify potential pathways linking CCA, psychiatric symptoms, resilience, and disaster-related experiences.

## 5 Conclusion

The current study is one of the few on CCA among individuals with clinical mental disorders. The results demonstrate that CCA is not merely a concern about the environment; instead, it is an expression of broad emotional and physiological distress and vulnerability emerging from the combination of emotional vulnerabilities (e.g., anxiety and somatization) and experiential exposure to disasters and climate change. For individuals with mental illness, CCA represents a new form of environment-related mental health problem that is closely associated with clinical psychiatric symptoms, thereby warranting clinical attention. Therapeutic approaches for addressing CCA should focus on anxiety and somatic symptoms, and strategies aimed to elevate levels of resilience may be likely effective.

## Data Availability

The raw data supporting the conclusions of this article are not publicly available due to privacy, confidentiality, and ethical restrictions under the IRB-approved protocol. Requests to access the data can be directed to the corresponding author.
